# Need for additional research on vestibular incision subperiosteal tunnel access (VISTA) technique in India

**DOI:** 10.1016/j.lansea.2023.100339

**Published:** 2023-12-08

**Authors:** Saravanan Sampoornam Pape Reddy, Delfin Lovelina Francis, Balaji Manohar, Shreehari Ambika Krishnan, Sukhbir Singh Chopra

**Affiliations:** a Department of Periodontology, Army Dental Centre (Research & Referral), Dhaula Kuan, Delhi, 110010, India; b Saveetha Dental College and Hospitals, SIMATS, Chennai, India; c Kalinga Institute of Dental Sciences, KIIT, Campus-5, Patia, Bhubaneswar, 751024, India

Gingival recession (also known as marginal tissue recession) was defined at the 2017 World Workshop in Periodontics as “the apical shift of the gingival margin with respect to the cemento-enamel junction”. Gingival recession (GR) is associated with attachment loss and exposure of the root surface to the oral environment.^1^ The global prevalence of GR for buccal gingival recessions was reported as 75.42% in 2022.^2^ Although GR can cause root sensitivity, root caries, and cosmetic issues, not all GR necessitate surgical intervention. However, untreated GR may result in progressive recession (odds ratio 2.43), which has a detrimental impact on the success of future treatment.^3^

The significance of GR as a public health concern is its reportedly high global prevalence. In addition, the aesthetic concerns with negative impact on smile, exposed root surfaces and an uneven gumline lead to self-consciousness and diminished self-esteem affecting social interactions and overall quality of life. The functional consequences include dentin hypersensitivity, increased caries risk and if untreated it can lead to further clinical attachment loss, tooth mobility finally leading to tooth loss. GR requires professional evaluation and appropriate treatment to manage the condition effectively.

Contemporary surgical techniques in the treatment of GR involve various approaches aimed at covering exposed root surfaces and improving the aesthetic appearance. These had a variety of approaches, beginning with a coronal to apical preparation using coronally advanced flap (CAF) or tunnel techniques that could be performed without a graft or as a bilaminar procedure with the addition of autogenous connective tissue graft (CTG), which remains the ‘gold standard' till date, or commercially available soft tissue substitutes. In addition, there are numerous autologous bioactive materials, such as plasma rich in growth factors, platelet rich fibrin (PRF), and xenogeneic materials, such as enamel matrix derivatives. Moreover, the techniques underwent an approach paradigm shift; where apical to coronal preparations have become increasingly popular in recent years as in vestibular incision subperiosteal tunnel access (VISTA) technique^4^ and pinhole surgical technique,^5^ with the goal of minimising soft tissue trauma and enhancing vascularity, resulting in improved root coverage outcomes.

Although VISTA technique was said to have been introduced in Europe in 2010 and published literature in 2011, it is now widely used in low-income and middle-income countries like India, especially in teaching institutions offering periodontology as a specialty. The VISTA technique is a surgical approach used for root coverage by placing a single vestibular incision and creating a subperiosteal tunnel to mobilise it passively and advance it coronally to cover the exposed roots of the teeth. The vestibular incision, as a single remote incision, reduces trauma to gingival margin while also providing access to insert any graft material or bioactive materials, conserving the integrity of gingival margins and interdental papillae. One of the primary advantages of this technique is its ability to achieve significant root coverage, particularly in GR cases of Cairo recession type 1 (RT1) (Miller Class I or II) or Cairo RT2 (Miller Class III) as the latter is more complex to treat. By creating a subperiosteal tunnel, access to the root surface is gained to perform procedures such as root planning, root surface biomodification and soft or hard tissue grafting. This allows for the coverage of exposed roots, improving aesthetics and reducing root hypersensitivity.^6^ The VISTA technique offers several benefits like reduced trauma to the soft tissues leading to decreased postoperative discomfort and faster healing in addition to being versatile. Additionally, the interdental papillae are preserved with this technique, leading to improved aesthetic outcomes due to maintained vascularity.^7^ The ability to customise the procedure based on the specific needs of each patient is a significant advantage.

Despite its advantages, it is important to note that, VISTA technique requires a high level of surgical skill and expertise. Proper patient selection, careful planning and precise execution are crucial for achieving optimal results. Additionally, as with any other root coverage surgery, the long-term stability of VISTA technique results may hinge on several variables, such as patient adherence to oral hygiene practices.^8^

While the available evidence suggests that the VISTA technique can be a promising approach for root coverage, it is important to interpret the findings with caution due to the limited and evolving nature of the evidence. Currently, the evidence available in India on VISTA technique predominantly consists of case reports, small scale retrospective studies and non-randomised trials, suggesting significant improvements in root coverage and aesthetics. However, a randomised controlled clinical trial (RCT)^9^ in India compared the applications of VISTA technique with CTG and PRF in 24 cases of Cairo RT2 GR, which resulted in significant mean root coverage (PRF group: 83%/CTG group: 86%) at six months follow-up. Though several studies report favourable outcome in terms of increased keratinised tissue width, reduction in recession and improved patient satisfaction, they do not provide the highest level of evidence at present.

As of May 2023, there is a limited number of RCTs evaluating the VISTA technique for root coverage, and their findings are still preliminary. To address the current gap in knowledge, well-designed, large-scale RCTs with long-term follow-up periods should be conducted to compare the outcomes of the VISTA technique with other established approaches for root coverage.^10^ This would provide more definitive conclusions regarding its effectiveness, reproducibility, and long-term stability.^11^ Dentists and periodontists should consider the available evidence, clinical expertise, and individual patient factors when determining the most appropriate treatment approach for root coverage.

## Contributors

Conceptualisation, formal analysis, methodology, writing–original draft: SSPR; Formal analysis, data curation, writing–revised draft: DLF; Project administration, visualisation, writing–final editing: BM; Supervision, reviewing: SAK; Validation, final reviewing: SSC.

## Declaration of interests

The authors declare no conflicts of interest.

## References

[1] Cortellini P., Bissada N.F. (2018). Mucogingival conditions in the natural dentition: narrative review, case definitions, and diagnostic considerations. J Periodontol.

[2] Yadav V.S., Gumber B., Makker K. (2022). Global prevalence of gingival recession: a systematic review and meta-analysis. Oral Dis.

[3] Chambrone L., Tatakis D.N. (2016). Long-term outcomes of untreated buccal gingival recessions: a systematic review and meta-analysis. J Periodontol.

[4] Zadeh H.H. (2011). Minimally invasive treatment of maxillary anterior gingival recession defects by vestibular incision subperiosteal tunnel access and platelet-derived growth factor BB. Int J Periodontics Restorative Dent.

[5] Reddy S.S.P. (2017). Pinhole surgical technique for treatment of marginal tissue recession: a case series. J Indian Soc Periodontol.

[6] Serry Y., Hegab M., Keraa K., El Barbary A. (2022). Platelet-rich fibrin versus connective tissue graft using vestibular incision subperiosteal tunnel access (VISTA) technique in multiple gingival recessions: randomized controlled trial. Perio J.

[7] Gil A., Bakhshalian N., Min S., Nart J., Zadeh H.H. (2019). Three-dimensional volumetric analysis of multiple gingival recession defects treated by the vestibular incision subperiosteal tunnel access (VISTA) procedure. Int J Periodontics Restorative Dent.

[8] de Sanctis M., Clementini M. (2014). Flap approaches in plastic periodontal and implant surgery: critical elements in design and execution. J Clin Periodontol.

[9] Hegde S., Madhurkar J.G., Kashyap R., Kumar M.S.A., Boloor V. (2021). Comparative evaluation of vestibular incision subperiosteal tunnel access with platelet-rich fibrin and connective tissue graft in the management of multiple gingival recession defects: a randomized clinical study. J Indian Soc Periodontol.

[10] Fernandez-Jimenez A., Estefania-Fresco R., Garcia-De-La-Fuente A.M., Marichalar-Mendia X., Aguirre-Zorzano L.A. (2021). Description of the modified vestibular incision subperiosteal tunnel access (m-VISTA) technique in the treatment of multiple Miller class III gingival recessions: a case series. BMC Oral Health.

[11] Belrhiti Z., Nebot Giralt A., Marchal B. (2018). Complex leadership in healthcare: a scoping review. Int J Health Policy Manag.

